# Infrarenal Remains Infrarenal—EVAR Suitability of Small AAA Is Rarely Compromised despite Morphological Changes during Surveillance

**DOI:** 10.3390/jcm11185319

**Published:** 2022-09-09

**Authors:** Corinna Becker, Tanja Bülow, Alexander Gombert, Johannes Kalder, Paula Rosalie Keschenau

**Affiliations:** 1 Department of Vascular Surgery, European Vascular Center Aachen-Maastricht, RWTH University Hospital Aachen, 52074 Aachen, Germany; 2 Institute of Medical Statistics, RWTH University Hospital Aachen, 52074 Aachen, Germany; 3 Department of Adult and Pediatric Cardiovascular Surgery, Universitätsklinikum Gießen und Marburg GmbH Standort Gießen, 35392 Gießen, Germany

**Keywords:** abdominal aortic aneurysm, endovascular aortic repair, endovascular grafting

## Abstract

The aim was to analyze small abdominal aortic aneurysm (AAA) morphology during surveillance with regard to standard endovascular aortic repair (EVAR) suitability. This retrospective single-center study included all patients (n = 52, 48 male, 70 ± 8 years) with asymptomatic AAA ≤ 5.4 cm undergoing ≥2 computed tomography angiography(CTA)/magnetic resonance imaging (MRI) studies (interval: ≥6 months) between 2010 and 2018. Aneurysm diameter, neck quality (shape, length, angulation, thrombus/calcification), aneurysm thrombus, and distal landing zone diameters were compared between first and last CTA/MRI. Resulting treatment plan changes were determined. Neck shortening occurred in 25 AAA (mean rate: 2.0 ± 4.2 mm/year). Neck thrombus, present in 31 patients initially, increased in 16. Average AAA diameters were 47.7 ± 9.3 mm and 56.3 ± 11.6 mm on first and last CTA/MRI, mean aneurysm growth rate was 4.2 mm/year. Aneurysm thrombus was present in 46 patients primarily, increasing in 32. Neck thrombus growth and neck length change, aneurysm thrombus amount and aneurysm growth and aneurysm growth and neck angulation were significantly correlated. A total of 46 (88%) patients underwent open (12/46) or endovascular (34/46) surgery. The planned procedure changed from EVAR to fenestrated EVAR in two patients and from double to triple fenestrated EVAR in one. Thus, standard EVAR suitability was predominantly maintained as the threshold diameter for surgery was reached despite morphological changes. Consecutively, a possibly different pathogenesis of infra- versus suprarenal AAA merits further investigation.

## 1. Introduction

Having been used for over 20 years, endovascular repair (EVAR) is nowadays not only an established therapy, it is often considered as the first-line treatment option for patients with abdominal aortic aneurysms (AAA) worldwide.

Compared to open surgical repair (OSR), it has a reduced short-time-mortality of approximately 1% (OSR 3–4%), but the advantage is lost in the long term [1]. When follow-up exceeds 8 years, there is even an increased aneurysm-related and overall mortality in EVAR patients compared to patients treated by OSR, which is mainly due to secondary sac rupture [2]. Relevant reintervention rates remain the main issue during follow-up and there is consensus in the national and international guidelines that lifelong surveillance is required in order to prevent post-EVAR complications [1,3,4].

Careful preoperative planning before EVAR is essential in order to achieve optimal results and special consideration has to be given to the proximal and distal landing zones [5,6,7,8]. In addition to patient specific factors, such as age and comorbidities, the evaluation of the proximal landing zone, i.e., the aneurysm neck, is the main criterium for choosing the type of aneurysm repair that can be offered. Mainly the diameter, length, shape, and angulation but also the presence of thrombus and/or calcification in the aneurysm neck determine whether infrarenal repair by means of EVAR is possible, more complex endovascular reconstructions (e.g., fenestrated/branched EVAR [FEVAR/BEVAR]) are required or whether open surgery (OSR) is the better alternative.

As for the indication for elective AAA repair, the maximum aneurysm diameter and the associated rupture risk, are the main criteria [1,3,4,9,10,11,12,13,14,15]. Additionally, aneurysm growth rate and possible symptoms have to be considered since they are associated with an increased rupture risk and therefore require intervention at smaller diameters. The current American and European Guidelines recommend surgery of asymptomatic aneurysms in men from a diameter of 5.5 cm [1,3,14] or a size increase of more than 10 mm per year [1,14]. Regarding women, there is a four-time higher risk of rupture so that elective AAA repair is recommended for women with an aneurysm of 5 cm or more in diameter [1,3,4,16].

There is currently no evidence showing a benefit of early repair of small asymptomatic aneurysms [15]. Therefore, small asymptomatic AAA (<5.5 cm in men or <5 cm in women) are monitored by regular ultrasound and/or cross-sectional imaging examinations until they reach the indication threshold or become symptomatic so that the risk of rupture surpasses the surgical risk [4,17].

However, some are still concerned that by waiting until the aneurysm reaches the diameter threshold for elective repair the possibility to perform infrarenal EVAR may be lost and the patient would have to undergo F/BEVAR or OSR. Earlier studies suggested that the morphology of infrarenal AAA, especially regarding the aneurysm neck, changes over time with a progression from a simple infrarenal pathology towards more proximal disease, thereby reducing the eligibility for EVAR [18,19].

Thus, this study was performed in order to analyze the morphological changes of infrarenal AAA, having a main focus on changes of the aneurysm itself and of the aneurysm neck over time, in all patients with small AAA (maximum diameter 5.4 cm) who had been referred to our department and admitted into the surveillance program. The aim was to evaluate whether the initial treatment plan had to be modified at the time the diameter threshold for aneurysm repair was reached.

## 2. Materials and Methods

This was a retrospective single-center study evaluating the computed-tomography angiography (CTA) or magnetic resonance imaging (MRI) imaging of vascular surgery patients with degenerative AAA. Additionally, basic demographic patient data including age, gender, and comorbidities were obtained. The study was approved by the local ethics committee (RWTH University Hospital Aachen, EK 186/16).

### 2.1. Definitions and Inclusion Criteria

The aorta was defined as aneurysmatic when the aortic diameter exceeded 30 mm [1,3,4,14,15]. The AAA were characterized as infra-, juxta- or suprarenal according to the current Guidelines of the European Society of Vascular Surgery (ESVS) [1]. Thus, an infrarenal AAA was defined as having a neck length ≥ 15 mm and being amenable to open surgical repair with infrarenal aortic clamp placement or endovascular aortic repair (EVAR) with a standard commercially available device; a juxtarenal AAA was defined as extending up to but not involving the renal arteries (neck length < 15 mm) and requiring suprarenal crossclamping during OSR or F/BEVAR for (durable) endovascular therapy; a suprarenal AAA was defined as extending up to the superior mesenteric artery, involving one or both renal arteries to be repaired and requiring supramesenteric or supraceliac crossclamping during OSR or F/BEVAR for endovascular therapy.

Indications for AAA repair were made according to the recommendations in the current guidelines of the ESVS [1] and of the German Society of Vascular Surgery [4]. Thus, 5.5 cm was considered the standard threshold diameter for elective AAA repair although female gender, rapid aneurysm growth rate, and occurrence of symptoms could necessitate earlier repair.

All AAA patients referred to our department between 2010 and 2018 who had undergone CTA or MRI of the aorta at least twice during this time period with a minimum time interval of six months between the exams and who had not yet undergone surgical AAA therapy were included.

The CTA/MRI imaging was analyzed by two independent examinators experienced in aortic measuring and sizing using the three-dimensional multiplanar reconstructions view mode (3D-MPR) of the OsiriX MD V.2.6 24-bit software (Picsmio SARL Bernex Switzerland).

**Figure 1:**
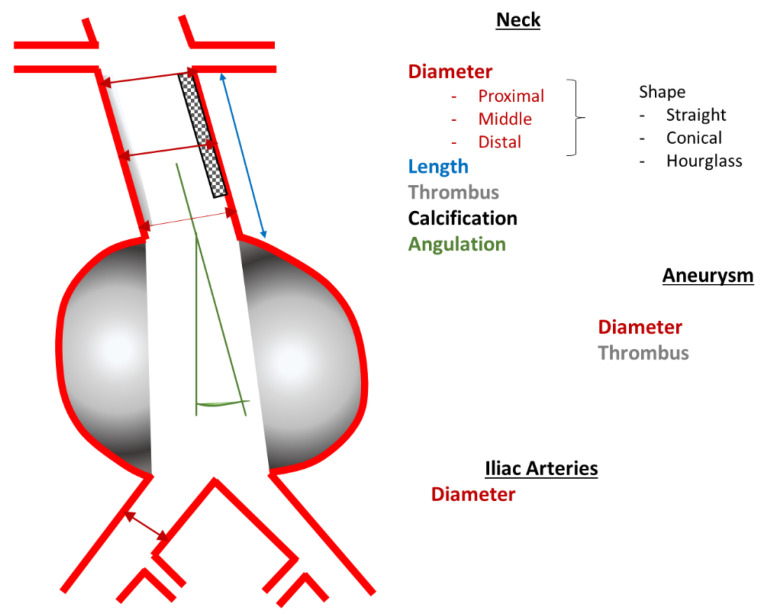
Parameters obtained from the three-dimensional imaging data.

The aim of the analysis was to evaluate whether the initial treatment strategy, that would have been recommended based on anatomical features of the proximal/distal landing zones, would have had to be changed over time. Thus, regarding the proximal landing zone, it was assessed whether an aneurysm ”remained“ suitable for infrarenal repair or whether degeneration of the proximal landing zone over time made a suprarenal repair necessary at the time the aneurysm reached the threshold diameter of 5.5 cm. As for the distal landing zones, it was assessed similarily whether, for instance, an additional iliac artery aneurysm repair was required at the time the AAA reached the threshold diameter for repair. Therefore, as depicted in Figure 1, the following parameters were measured: the maximum aneurysm diameter; the length, diameter, angulation, and quality (presence of thrombus and/or calcification, shape) of the proximal landing zone and the diameter of the distal landing zone. A detailed presentation of the analyzed parameters is given in Table 1 and exemplary measurements are shown in Supplementary Figures S1–S6 in Supplementary Materials. Two examinators consenting the values performed all measurements.

In all patients, the above-mentioned parameters and resulting surgical approaches were compared between the first and the last available CTA/MRI study before surgical repair or, if untreated, before the end of the study period.

The surgical approaches considered were open repair (OSR), infrarenal endovascular aortic repair (EVAR) and fenestrated/branched EVAR (FEVAR/BEVAR). The recommendation of the type of aortic repair based on the parameters measured in the CTA/MRI scan was made by two experienced vascular surgeons familiar with both open and endovascular aortic surgery. Then, the decision for the type of aneurysm repair, especially between endovascular and open repair, was individually discussed and decided in an aortic conference.

### 2.2. Exclusion Criteria

Patients with thoracoabdominal aneurysms, post-dissection aneurysms, anastomotic and infectious aneurysms. Further, patients were excluded who underwent AAA repair for any reason before having a second CTA/MRI scan and patients in whom the last available CTA/MRI scan was less than 6 months after the first were also excluded.

### 2.3. Statistical Analysis

The statistic evaluation was performed with Microsoft Office 365 ProPlus Excel (Microsoft Corporation, DC, USA) and SAS 9.4 (SAS Institute Inc., Cary, NC, USA). Categorical parameters are given in absolute numbers and percentages, continuous parameters in mean and standard deviation. Correlations were determined using correlation coefficients of Pearson and Spearman with a 95% confidence interval (α = 0.05).

For the correlation analysis, infra- and juxtarenal AAA were considered together, so that two groups, “AAA with neck” and “no-neck-AAA” could be examined.

In the group “no-neck-AAA” the correlation between thrombosis in neck and aneurysm, between the angulation and the aneurysm growth, between aneurysm growth, and aneurysm thrombus growth was analyzed.

In the group “AAA with neck” correlation between the change of the neck length and the thrombus size in neck in the first CTA, the correlation between thrombus growth in the neck and the change of the neck length, between the amount of aneurysm thrombus and aneurysm growth and between the aneurysm growth and change of the neck angulation was determined.

## 3. Results

**Figure 2:**
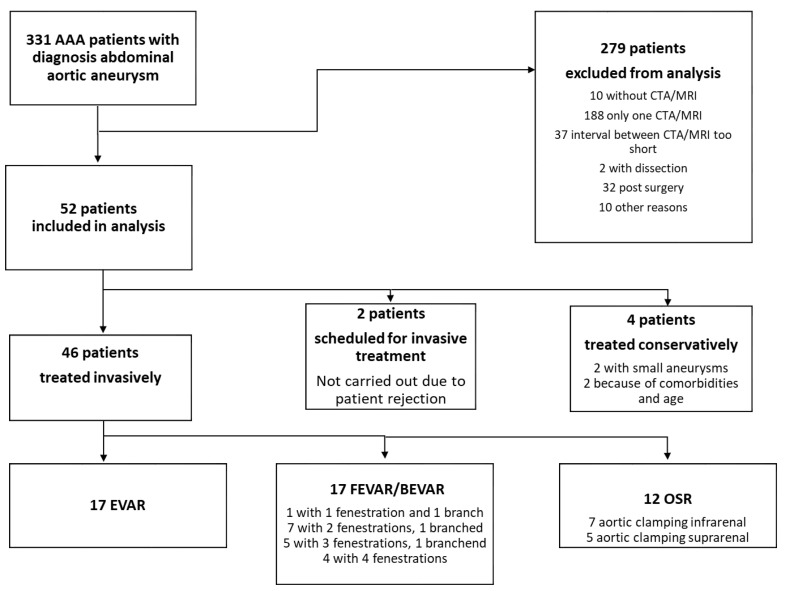
Flow chart showing the inclusion/exclusion of patients in the present study. AAA: abdominal aortic aneurysm, EVAR: endovascular aortic repair, FEVAR: fenestrated EVAR, BEVAR: branched EVAR, OSR: open surgery, CTA: computed tomography angiography, MRI: magnetic resonance imaging.

Out of 331 AAA patients diagnosed with abdominal aortic aneurysm during the study period in our department, only 52 met the inclusion/exclusion criteria (Figure 2). As tertiary aortic center, the first patient contact took place as surgery was indicated. Therefore, almost no preoperative CT or MRI scan was available. In addition, it is a common practice in our patient cohort to screen AAA only with ultrasound until the indication size is reached.

46 patients (88%) were male. The basic demographic patient data including comorbidities are given in Table 2. The mean age at the time of the first examination was 70 ± 8 years. The mean age at the time of the last examination was 73 ± 8 years. The average time interval between two examinations were 19.5 ± 21 months. The average time interval between first and last examination was 32 ± 29 months. As 50 patients received CTA scans and only 2 patients MRI scans, no statistical analysis were performed between these two examination techniques.

### 3.1. Analysis of Morphological Parameters

Table 3 demonstrates the results of morphological parameter measurements in the first and last CTA, respectively.

Initially, 35 AAA were classified as “with neck” and 17 as “no-neck” AAA. As for the aneurysm neck shape in the first CTA/MRI scan, 2 necks were conical, 1 shaped as an hourglass, and the remaining 32 aneurysms had straight necks. In the last CTA/MRI scan, 3 necks were conical, 1 shaped as an hourglass, and 31 aneurysms had straight necks.

The mean angulation between neck and aneurysm was 27.0° ± 18.99° on the first CTA/MRI scan and 34.6° ± 23.75° on the last CTA/MRI scan. The angulation increased in 41 patients (79%), remained constant in 3 patients (6%), and decreased in 8 patients (15%).

In 23 of the AAA “with neck” an absolute neck shortening of 3.7 ± 6.2 mm was observed, corresponding to a shortening rate of 2.0 ± 4.2 mm per year. Aneurysm neck thrombus was present in 31 patients in the first CTA, neck thrombus and/or calcification in 38 patients. In 6 cases, the neck thrombus remained constant, in 9 cases it decreased and in 16 cases it increased. One patient developed new neck thrombus over time. The mean growth of aneurysm neck thrombus was 5% (−29% up to +57%).

The average AAA diameter was 47.7 ± 9.3 mm at the time of the first and 56.3 ± 11.6 mm at the time of the last CTA/MRI scan. The mean aneurysm growth rate was 4.20 mm/year (0–20.18 mm/year). Aneurysm thrombus was present in 46 patients in the first CTA. It increased in 32 patients, remained constant in 25, and decreased in 9 patients. In one case a new aneurysm thrombus had developed at the time of the last CTA. The mean thrombus growth in the aneurysm was 8% (−19% up to +52%).

In 18 cases (35%), the common iliac arteries were dilated to more than 20 mm of which 8 (15%) were bilateral and 10 (19%) were unilateral, with the right side more frequently affected.

A total of 11 patients had no calcification of the common iliac arteries, 39 had calcification with stenosis, and 1 had calcification without stenosis. In one case, it was not possible to determine the calcification due to image quality. At the time of last CTA/MRI, three additional patients had developed common iliac artery calcification without stenosis.

### 3.2. Correlation Analysis

In the “no neck” group, there was no significant correlation between thrombus growth in neck and aneurysm (r(Pearson) = 0.31, *p* = 0.23; r(Spearman) = 0.33, *p* = 0.19), between the angulation and the aneurysm growth (r(Pearson) = 0.26, *p* = 0.32) or between aneurysm growth and aneurysm thrombus growth (r(Pearson) = −0.27, *p* = 0.29).

In the group with neck, there was no significant correlation between the change of the neck length and the thrombus size in neck in the first CTA (r(Pearson) = −0.31, *p* = 0.07). However, there was a significant correlation between thrombus growth in the neck and the change of the neck length (r(Spearman) = 0.37, *p* = 0.03). Further, there were significant correlations between the amount of aneurysm thrombus and aneurysm growth (r(Pearson) = 0.39, *p* = 0.02 r(Spearman) = 0.45, *p* = 0.007) as well as between the aneurysm growth and change of the neck angulation (r(Pearson) = 0.47, *p* = 0.004 r(Spearman) = 0.41, *p* = 0.01). Analyzing the data set to explore possible additional affecting factors for proximal neck changes over time, medication and age marked no significant values.

### 3.3. Types of Operative Therapy

A total of 46 of the 52 patients (88%) underwent surgery (open or endovascular reconstruction) during the study period after having had at least two imaging scans. Out of these, 12 patients (26%) underwent OSR, 17 (37%) EVAR, and 17 (37%) FEVAR/BEVAR. 4 patients were treated conservatively (2 small aneurysms and 2 because of age and comorbidities). In total, 2 patients were scheduled for surgery, but it was not performed because of patient rejection. In the case of the smallest aneurysm (38 mm at the time of the second CTA), the indication for surgery was given due to an aneurysm of the right common iliac artery, with a diameter of 37 mm. In this case, an endovascular treatment with an iliac side branch was discussed and discarded, as the access vessel anatomy and the additional vascular occlusive disease supported the decision for an open aorto iliac repair. Consequently, the patient was treated by an open aorto-biiliac graft with selective bypass to the left internal iliac artery and ligation of the right internal iliac artery.

### 3.4. Procedural Changes

**Figure 3:**
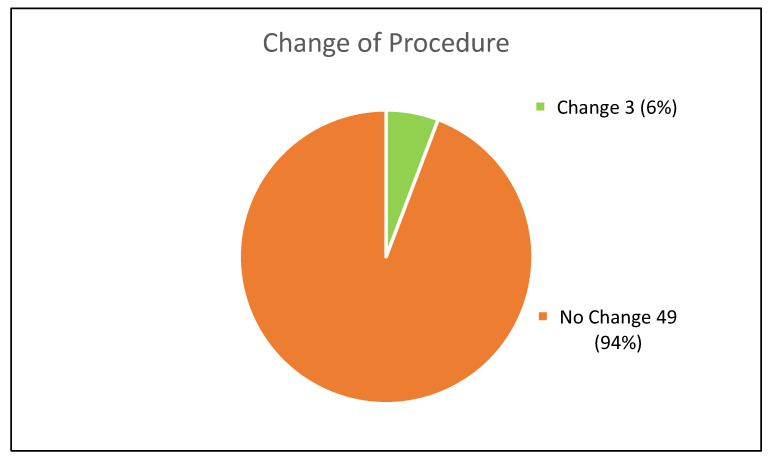
Change of Procedure.

There has been a procedural change in three cases (Figure 3). In one case, the planned treatment changed from EVAR to FEVAR because the neck shortened from 24 mm to 0 mm. In the second case, it was a “no neck” AAA from the beginning, so that FEVAR was planned, but it changed from two fenestrations to three. In the third case, an EVAR would have been possible initially, but due to an increase in neck thrombus FEVAR was deemed more adequate later on.

## 4. Discussion

The present study demonstrates that in the majority of patients with small infrarenal AAA, the aneurysm morphology remains suitable for standard EVAR at the time the threshold diameter for operative AAA repair is reached. Although there are anatomical changes over time, including aneurysm growth, increasing neck angulation, an increase in thrombus burden in the aortic neck as well as in the aneurysm itself, they do not influence the clinical decision-making with regard to the choice of infrarenal versus suprarenal repair. Further, our results hint at a different pathophysiological behavior of AAA with neck and “no-neck” aneurysms over time, which may be associated with a different role of the thrombus burden in these two entities.

A recent Cochrane meta-analysis of four randomized controlled trials found no evidence for the treatment of small (4.0–5.4 cm) asymptomatic AAA either by OSR or by EVAR [17]. However, since the trials included in the meta-analysis—the Aneurysm Detection And Management (ADAM) trial, the United Kingdom Small Aneurysm Trial (UKSAT), the Comparison of surveillance versus Aortic Endografting for Small Aneurysm Repair (CAESAR) trial and the Positive Impact of endoVascular Options for Treating Aneurysms earLy (PIVOTAL) trial—date back several years and an improved risk-to-benefit ratio may nowadays be assumed especially for EVAR, the discussion of whether or not to treat AAA below the general threshold diameter of 5.5 cm keeps re-emerging. In this context, there is discussion about a possible decline in suitability for EVAR over time, so that earlier repair might prevent patients from more extensive (suprarenal) endovascular or open surgery.

An earlier study analyzing changes of the aneurysm neck morphology of small AAA over time in computed tomography studies found that the aneurysm neck length decreases approximately 1 mm per year, while the neck diameter increases only approximately 0.25 mm per year [18]. As a result, 8% of the patients became unsuitable for standard EVAR at the time the threshold diameter for aneurysm repair was reached. Although in the present study a faster average neck shortening of 2 mm ± 4.2 mm per year was observed in 66% (23/35) of the infrarenal AAA, this did not impact the suitability for EVAR. Similarly, Yau et al. reported statistically significant changes in aortic neck length, diameter and suprarenal angulation over time in a cohort of 54 patients but found that this did not influence the suitability for EVAR as per instructions for use (IFU) during a median follow-up of 24 [15,16,17,18,19,20,21,22,23,24,25,26,27,28,29,30,31,32,33,34,35,36] months [20]. The authors concluded that most AAA that are initially infrarenal remain infrarenal, suggesting that the differentiation between supra- and infrarenal aneurysmatic degeneration of the aorta is determined very early in AAA pathogenesis [20]. Supporting this hypothesis, their results were recently confirmed in a larger retrospective multicenter analysis in which 98% of patients were still suitable for EVAR after 2 years, with aneurysm neck shortening being the main reason for becoming unsuitable for EVAR in the remaining 2% of patients [21].

Accordingly, the initial treatment plan did not change in the vast majority of patients in the present study. One patient of the herein described cohort became unsuitable for infrarenal EVAR due to neck shortening, the other was deemed unsuitable due to an increase in neck thrombus. In fact, although aortic neck thrombus was not present in all patients, we found a significant correlation between the change in aortic neck length and neck thrombus growth in percentage of the circumference among the AAA “with neck” as opposed to the “no neck” group. Only one of the aforementioned studies assessed the presence of aortic neck thrombus and/or calcification without finding a significant change in the amount over time [20] and an association between the amount of neck thrombus and neck diameter has been previously described [22]. However, a possible correlation between aortic neck shortening and neck thrombus burden and/or increase has not been investigated before to our knowledge.

Aneurysm thrombus, also called intraluminal thrombus (ILT), on the other hand has been the subject of multiple studies. Although there is consensus that ILT is of importance with regard to AAA pathogenesis, growth rate, and rupture risk; the exact pathophysiology is still unclear [23]. It is recognized that ILT in AAA is biologically active and can contribute to aneurysm wall weakening by inducing proteolysis and activating matrix metalloproteinases [23,24,25]. Thus, it has been found in an experimental study that ILT precedes AAA growth [26], and the majority of clinical studies have found a correspondence between ILT presence and increased AAA growth rate [27], at least up to a certain AAA size [23]. This is in accordance with the present study results, showing a positive correlation between the amount of AAA thrombus and AAA growth in small AAA. However, there is also the seemingly conflicting assumption that thicker ILT may be protective by reducing wall stress [28] and thereby slowing AAA growth [29]. Probably, ILT thickness and distribution have to be examined more closely in order to determine whether it is protective or harmful [30,31,32], but this was not investigated in the present study. Further, it is still not entirely clear why ILT formation in AAA seems to have other pathophysiological consequences than in other regions, e.g., popliteal artery aneurysms [23] or in the aneurysm neck.

Interestingly, it has been observed in another study that AAA with a short neck (<15 mm) have higher relative ILT volumes and a higher estimated rupture risk in the finite element analysis compared to those with longer neck [33]. Considering, firstly, the above-mentioned differences between infra- versus suprarenal AAA regarding the neck behavior and, secondly, the preservation of EVAR suitability over time, we support the hypothesis that infra- and suprarenal AAA are likely two different entities, which can have a different natural course. Considering the known histological differences with an altered elastin-to-collagen-ratio of the infrarenal compared to the suprarenal aorta [34], this would only be logical, but further multimodal investigations, ideally combining fundamental and clinical research, are required to verify this assumption.

The present study has several limitations, firstly the retrospective approach and the small number of patients considering the long study period. Although data from 356 patients with a diagnosed AAA were available, only 52 of these patients could be included in the present analysis, mostly due to the lack of a second CTA/MRI scan. In most cases, this was due to insufficient patient compliance, which is a known problem in vascular surgery patients in daily practice.

Further, selection bias is possible due to the fact that many special cases are treated in our center, i.e., AAA in a patient with a pelvic kidney and the “ordinary AAA” are treated in smaller hospitals. Therefore, the results may not be transferable to all AAA patients. Finally, due to the important evolvement of endovascular techniques and the increased experience with complex endovascular procedures over the study period, treatment strategies have changed in general so that aortic surgeons are nowadays more likely to choose FEVAR over infrarenal EVAR for endovascular repair of a AAA with a borderline neck. Further, as was the case in one of the patients in this study, four fenestrations are chosen more often over a lesser number of fenestrations in anticipation of proximal disease progression and in order to achieve increased durability. Finally, although the quality of the distal landing zones, i.e., the common iliac arteries (CIA), is undoubtedly relevant in the planning of aortic aneurysm repair [35] and may even influence preoperative AAA growth [36], CIA morphology was not the main focus of the present work and thus not investigated or discussed in detail.

## 5. Conclusions

Although there are anatomical changes of the aneurysm and aneurysm neck over time during surveillance of small infrarenal AAA, the suitability for standard EVAR is maintained in the majority of cases. Thus, the present study supports the theory that there is a different pathogenesis of juxta- or suprarenal AAA versus infrarenal AAA. This should be investigated in further analyses with special regard to the role of aneurysm/aneurysm neck thrombus.

## Figures and Tables

**Table 1:** Parameters obtained from the three-dimensional imaging data.

Proximal Landing Zone, i.e., Aneurysm Neck	Shape	straight, conical, hourglass, kinked
	Diameter [mm]:	outer wall to outer wall - proximally (at the level of the most caudal renal artery) - in the middle - distally In the case of juxtarenal aneurysms, only the diameter at the level of the renal arteries
	Length [mm]	distal renal artery to aneurysm
	Morphology	- thrombosis in percent of the circumference [%] - presence of calcification [yes/no, %]
	Angulation [°]	between neck and aneurysm
Aneurysm	Type	- with neck (infra-/juxtarenal) - ”no neck“ (suprarenal)
	Maximum diameter [mm]	outer wall to outer wall
	Thrombus	area in percent of the cross-section [%]
Distal Landing Zone, i.e., Common Iliac Arteries	Maximum diameter [mm]	outer wall to outer wall

**Table 2:** Basic patient characteristics.

**Age**	**Mean ± SD (Years)**
First CTA/MRI ^1^	70 ± 8
Last CTA/MRI ^1^	73 ± 8
**Sex**	**Patient number [*n* (%)]**
Male	46 (88)
Female	6 (12)
**Comorbidities**	
Arterial hypertension	37 (71)
Coronary heart disease	34 (65)
Peripheral arterial occlusive disease	17 (33)
Cerebrovascular pathologies	11 (21)

^1^ CTA: computed tomography angiography; MRI: magnetic resonance imaging; SD: standard deviation.

**Table 3:** Results of morphological parameter measurements.

	First CTA/MRI ^1^	Last CTA/MRI ^1^
	Mean ± SD [mm]	Mean ± SD [mm]
Maximum aneurysm diameter	47.7 ± 9.3	56.3 ± 11.6
	patient number [*n* (%)]	patient number [*n* (%)]
Aneurysm type		
with neck	35 (67)	33 (63)
“no neck“	17 (33)	19 (36)
Neck shape		
straight	28 (54)	26 (50)
Conical	2 (4)	3 (6)
Hourglass	1 (2)	1 (2)
Bended	4 (8)	5 (10)
Neck thrombus present	31 (60)	32 (63)
Increased	N.A. ^2^	16 (31)
Unchanged	N.A. ^2^	6 (12)
Decreased	N.A. ^2^	9 (17)
New	N.A. ^2^	1 (2)
Aneurysm thrombus present	46 (88)	47 (90)
Increased	N.A. ^2^	32 (61)
Unchanged	N.A. ^2^	5 (10)
Decreased	N.A. ^2^	9 (17)
New	N.A. ^2^	1 (2)
Angulation		
Increased	N.A. ^2^	41 (79)
Unchanged	N.A. ^2^	3 (6)
Decreased	N.A. ^2^	8 (15)
Diameter common iliac arteries > 20 mm		
Overall	18 (35)	18 (35)
Unilateral	10 (19)	9 (17)
Bilateral	8 (15)	9 (17)

^1^ CTA: computed tomography angiography; MRI: magnetic resonance imaging; ^2^ N.A.: not applicable.

## Data Availability

The data underlying this study are available on request from the first and corresponding authors. The data are not publicly available due to restrictions resulting from the EU general data protection regulation.

## Supplementary Materials

The following supporting information can be downloaded at: https://www.mdpi.com/article/10.3390/jcm11185319/s1, Figure S1: Aneurysm neck, length and juxtarenal diameter; Figure S2: Aneurysm neck, thrombosis; Figure S3: Aneurysm neck, middle diameter; Figure S4: Aneurysm neck, distal diameter; Figure S5: Aneurysm, maximum diameter and thrombosis; Figure S6: Right common iliac artery, diameter.
