# Supplementary figures and images for: Infrarenal Remains Infrarenal—EVAR Suitability of Small AAA Is Rarely Compromised despite Morphological Changes during Surveillance
Source: J Clin Med. 2022 Sep 9;11(18):5319. doi: 10.3390/jcm11185319 (PMC9501454; doi:10.3390/jcm11185319)

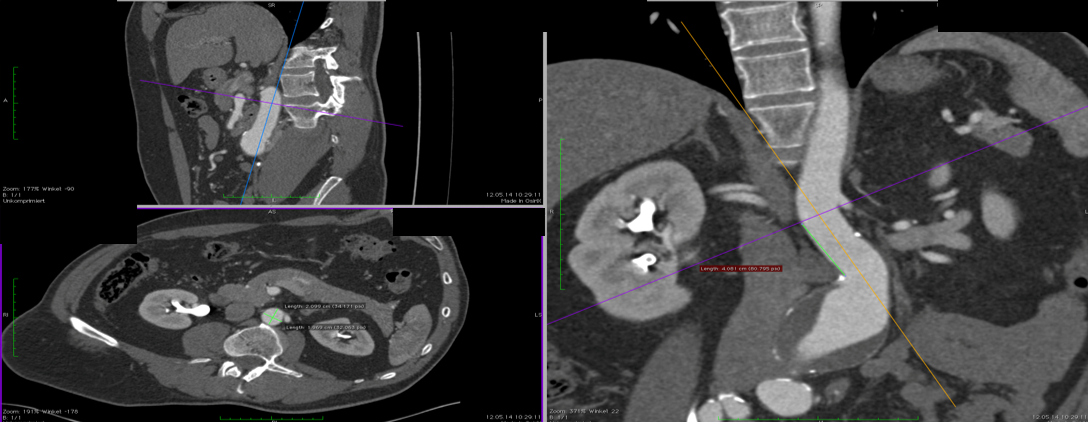

Supplement: Supplementary file 1 [file jcm-11-05319-s001.zip › Figure S1.tif]

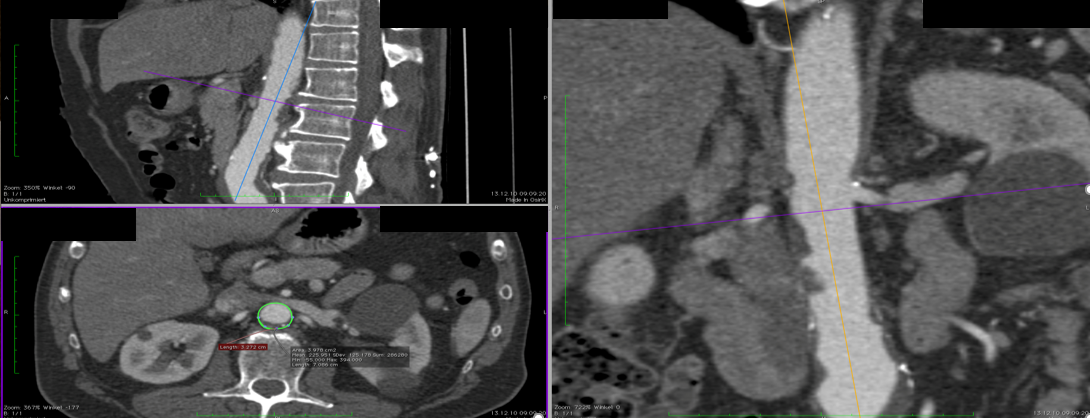

Supplement: Supplementary file 1 [file jcm-11-05319-s001.zip › Figure S2.tif]

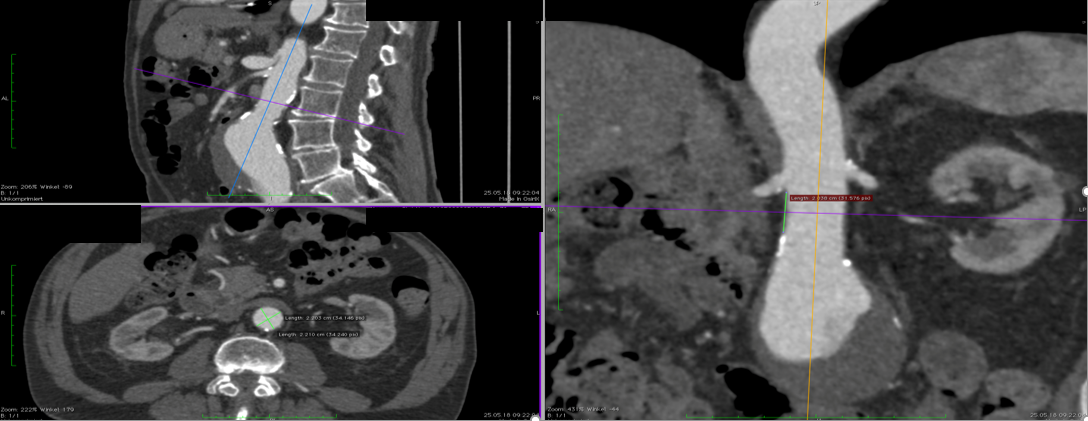

Supplement: Supplementary file 1 [file jcm-11-05319-s001.zip › Figure S3.tif]

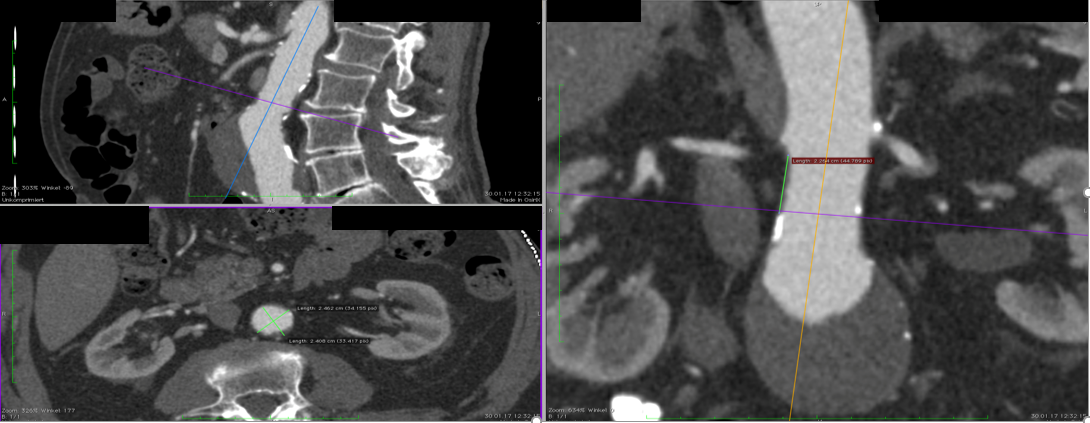

Supplement: Supplementary file 1 [file jcm-11-05319-s001.zip › Figure S4.tif]

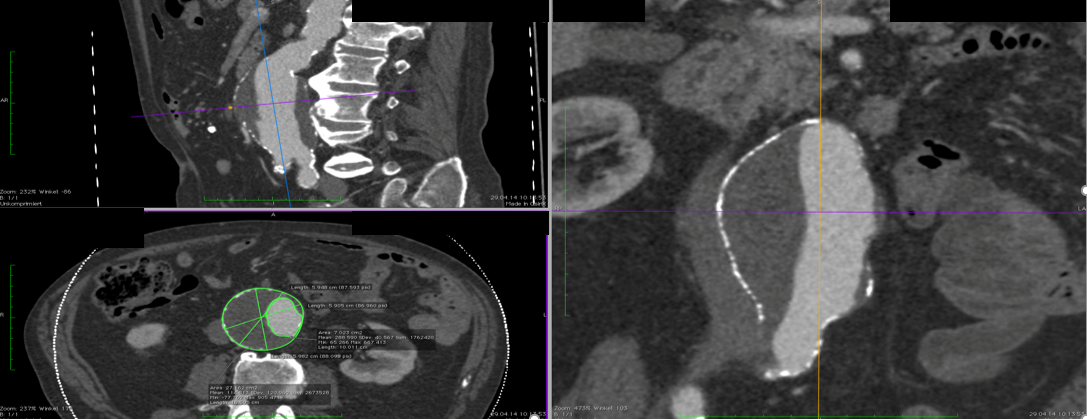

Supplement: Supplementary file 1 [file jcm-11-05319-s001.zip › Figure S5.tif]

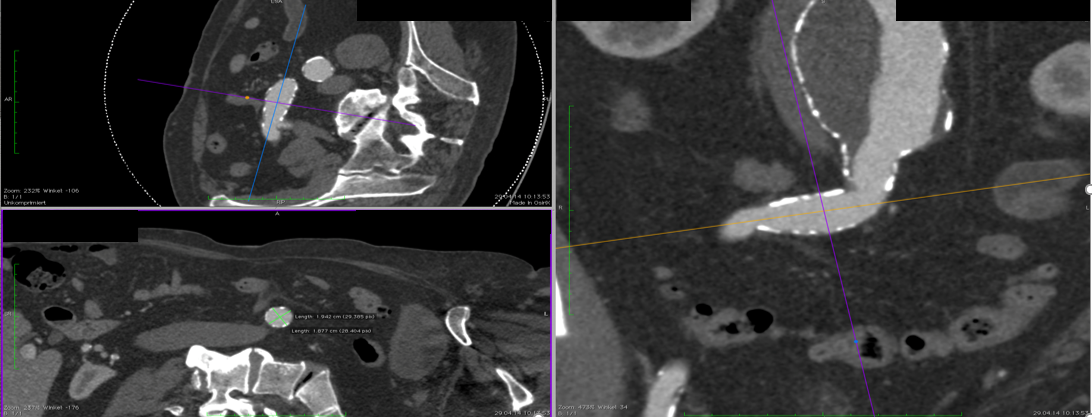

Supplement: Supplementary file 1 [file jcm-11-05319-s001.zip › Figure S6.tif]
